# Data-Driven Models of Efficient Chromatic Coding in the Outer Retina

**DOI:** 10.1523/ENEURO.0397-22.2022

**Published:** 2022-12-12

**Authors:** Luisa Ramirez, Ronald Dickman

**Affiliations:** 1 Departamento de Física, Universidade Federal de Minas Gerais, Belo Horizonte, Minas Gerais CEP 31270-901, Brazil; 2 National Institute of Science and Technology for Complex Systems, Belo Horizonte, Minas Gerais CEP 31270-901, Brazil

## Abstract

Recent experimental work on zebrafish has shown the *in vivo* activity of photoreceptors and horizontal cells (HCs) as a function of the stimulus spectrum, highlighting the appearance of chromatic-opponent signals at their first synaptic connection. Altogether with the observed lack of excitatory intercone connections, these findings suggest that the mechanism yielding early color opponency in zebrafish is dominated by inhibitory feedback. We propose a neuronal population model based on zebrafish retinal circuitry to investigate whether networks with predominantly inhibitory feedback are more advantageous in encoding chromatic information than networks with mixed excitatory and inhibitory mechanisms. We show that networks with dominant inhibitory feedback exhibit a unique and reliable encoding of chromatic information. In contrast, this property is not guaranteed in networks with strong excitatory intercone connections, exhibiting bistability. These findings provide a theoretical explanation for the absence of excitatory intercone couplings in zebrafish color circuits. In addition, our study shows that these networks, with only one type of horizontal cell, are suitable to encode most of the variance from the zebrafish environment. However, at least two successive layers of inhibitory neurons are needed to reach the optimum. Finally, we contrast the encoding performance of networks with different opsin sensitivities, showing an improvement of only 13% compared with zebrafish, suggesting that the zebrafish retina is adapted to encode color information from its habitat efficiently.

## Significance Statement

Recent experiments show that outer retinal circuits in zebrafish exhibit color opponency at the first synaptic contact between cones and HCs. We propose a neuronal population model to study dynamical and mechanistic properties of zebrafish-like networks in the context of efficient chromatic coding. We show that networks with strong excitatory feedback can lead to an ambiguous color encoding, providing a plausible explanation for the primarily inhibitory feedback observed in zebrafish. Finally, we parametrize the network coupling parameters to investigate network architectures allowing an efficient chromatic coding of the zebrafish habitat. Our findings suggest that retinal networks with red, green, and blue zebrafish cones are highly efficient in encoding such natural spectral information. Still, further improvement is possible with additional inhibitory feedback at downstream layers.

## Introduction

The study of chromatic information processing in the retina has a long history (Kolb, 1994; Cronin et al., 2014; Nelson, 2017; Baden and Osorio, 2019). Still, some questions on the diversity and functionality of retinal circuits remain unanswered (Baden and Osorio, 2019; Thoreson and Dacey, 2019). Information theory has provided insights into these questions via principles of efficient information transmission (Cover, 1999), related to reliable encoding and transmission of spectral information via neuronal responses and synaptic interactions at a minimum energetic cost (Lee et al., 2002; Kelber and Osorio, 2010; Zimmermann et al., 2018). More specifically, we identify efficient coding processes as those maximizing the information transfer rate over the limited retinal capacity (Attneave, 1954; Buchsbaum and Gottschalk, 1983; Cover, 1999), for instance, by adapting to the statistics of natural stimuli.

Retinal circuits are organized in layers, such that the outermost neurons receive photonic stimuli, while the innermost neurons connect to downstream brain circuits. Photoreceptor neurons, specifically cones, are the basis of color vision at photopic conditions. They are categorized by their independent spectral response via the sensitivity function, which displays a vast diversity across species (Cronin et al., 2014). In retinas, functional cone circuits determine the color-space, defined by the number of functional chromatic channels (Kelber and Osorio, 2010; Cronin et al., 2014). For instance, human cones, sensitive to long-wavelength, middle-wavelength, and short-wavelength stimuli, shape a trichromatic visual system. Responses from the photoreceptor layer activate horizontal cells (HCs), following the retinal pathway. Similarly to photoreceptors, the diversity of these neurons across species is vast and their functionality has been extensively investigated (Chapot et al., 2017). Broadly, HCs connect laterally to several photoreceptors, integrating spatially localized responses that are further processed by downstream retinal layers.

While the selective advantage conferred by color vision derives from object discrimination via spectral contrast (as opposed to mere brightness contrast), the response curves of different type of opsins have significant overlap, yielding highly redundant signals. Reducing such redundancies enhances the transfer rate of color information optimizing the chromatic encoding process (Barlow, 1961). For instance, theoretical work by Buchsbaum and Gottschalk (1983) on trichromatic visual systems shows that given the covariance matrix of photoreceptor responses, the eigenvalue or principal component transformation is optimal at decorrelating these three chromatic channels, resulting in a more efficient information encoding. Such a transformation leads to the appearance of chromatic opponent signals, also predicted by color-opponency theory (Shevell and Martin, 2017), meaning that cone responses turn inhibitory in certain ranges of the spectrum to guarantee their linear independence. Over the past decades, experimental work in vertebrates (De Valois et al., 1966; Patterson et al., 2019) has shown color opponency in retinal-ganglion layers and downstream neuronal circuits. Evidence of opponency in outermost retinal circuits, however, is more recent (Packer et al., 2010; Yoshimatsu et al., 2021a) and suggests that cone spectral responses turn opponent at the first synaptic contact with HCs.

*In vivo* recordings of zebrafish cones in response to chromatic stimuli (Yoshimatsu et al., 2021a) show evidence of color-opponent circuits in outer retinal layers, yielding important insights into retinal color processing. Such work highlights the importance of interneurons in color encoding, showing that these opponent responses depend on inhibitory feedback from mostly one type of HC, whereas excitatory intercone connections are negligible. These experimental observations together with ideas of efficient coding suggest that color processing in zebrafish might be optimized at the earliest retinal stage. Moreover, such cone responses follow qualitatively the principal components of hyperspectral natural images, suggesting that zebrafish circuits are adapted to their natural chromatic statistics. Following these experimental results, we ask whether (1) these findings can be generalized at the level of network dynamics, leading to a broader understanding of retinal circuits for color discrimination; and (2) whether we can contrast the color encoding performance of zebrafish circuits with other networks sharing similar architectures.

To address these questions, we propose a retinal population model to investigate chromatic encoding. First, we investigate the role of excitatory and inhibitory synaptic connections in dichromatic and trichromatic networks with structures similar to that of zebrafish. We study whether the absence of excitatory intercone connections facilitates coding of chromatic information, providing a plausible explanation for the predominant inhibitory feedback mechanism observed in zebrafish (Yoshimatsu et al., 2021a). Second, we include the statistics of hyperspectral images of zebrafish environments (Cornelius, 2021), quantifying the encoding performance of the previously investigated networks. More specifically, we study the limitations of zebrafish-like networks to capture all the accessible chromatic variance. As previously discussed, experiments in zebrafish show that only one type of HC, from the four identified, contribute to the chromatic-opponent responses of cones. Similarly, the results suggest that the ultraviolet (UV) channel is independent from the other three channels (red, green, and blue), being unaffected by HC activity. Consequently, in this work, we focus in retinal networks with (1) combinations of red, blue, and green photoreceptors, and (2) inhibitory feedback from one type of HC. Species with early chromatic opponency but different retinal architectures, such as butterflies (Belušič et al., 2021), are left as topics for future work.

## Materials and Methods

### Dynamics of outer retinal networks

**Figure 1.**
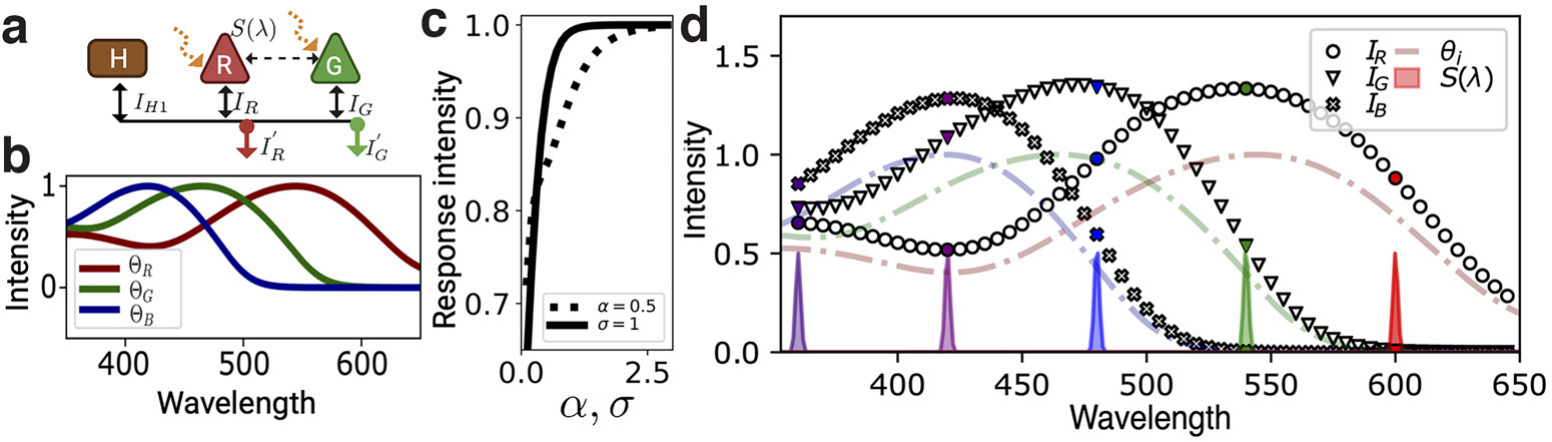
***a***, Dichromatic sketch of a fully connected network with an external stimulus, *S*(*λ*). R: red cones, G: green cones, B: blue cones, H: horizontal cells. Solid black arrows represent inhibitory synaptic connections on cones from horizontal cells. Dashed black arrows represent excitatory synaptic connections between cones. ***b***, Sensitivity functions of independent red, green, and blue zebrafish opsins (Cornelius, 2021; Yoshimatsu et al., 2021b). ***c***, Cone response in Equation 1 as a function of the free parameters in Equation 2; the solid (dashed) line corresponds to the variation of the intensity (SD) for a fixed value *σ* = 1 (*α* = 0.5). ***d***, Independent responses of photoreceptors to narrow Gaussian stimuli. Dashed curves are red, green, and blue zebrafish sensitivity curves and colored distributions correspond to five Gaussian stimuli with *α* = 0.5 and SD *σ* = 1 nm. Markers correspond to the independent responses, described by Equation 1, of the three photoreceptors. Colored markers show the response to the five plotted stimuli.

We begin by studying the dynamic properties of dichromatic and trichromatic networks with photoreceptors sensitive to short (B), middle (G), and long (R) spectral ranges, interacting via direct intercone excitatory couplings or indirect HC-cone inhibitory couplings. Figure 1*a* shows an example of a typical R-G dichromatic network with inhibitory feedback, that is, with HC-cone synaptic connections. More precisely, light stimuli induce linear excitatory responses, *I_i_*, of photoreceptor neurons (R and G), which in turn activate horizontal cells (H). The integrated response of horizontal cells, *I_H_*, provides an inhibitory feedback to photoreceptor neurons, inducing final responses, $I_{i}^{\prime }$, sent to downstream retinal circuits. Networks with excitatory intercone connections and inhibitory feedback have additional cone-cone connections (Fig. 1*a*, dashed arrow). We denote networks with dominant inhibitory feedback and weak intercone connections as Type I; networks with strong excitatory intercone connections are denoted Type II.

One way of characterizing visual stimuli is via the spectral density, *S*(*λ*), which contains all relevant chromatic information. To calculate the magnitude of the isolated cone response, *I_i_*, to a stimulus with spectral density, *S*(*λ*), we integrate the product of Θ*_i_*(*λ*), the corresponding sensitivity function, and *S*(*λ*), over the spectrum:

(1) $$I_{i}=\tanh\left(\int \Theta_{i}(\lambda )\;S(\lambda )\;d\lambda \right).$$

Determining responses to specific wavelength intervals requires stimuli with a narrow spectral distribution, conveniently represented by Gaussian distributions centered at a characteristic wavelength *λ*_0_, and having a small SD, *σ*, that is,

(2) S(λ)=α exp(−(λ−λ0)22σ2),

with *α* the stimulus intensity. Figure 1*c* shows the response in Equation 1 as a function of the intensity and SD in Equation 2 for a fixed value *λ*_0_. We see that for highly intense or spectrally broad stimuli, cone responses saturate as expected. To avoid this saturation regime, we use the parameters *σ*_0_ = 1 nm and *α* = 0.5 to characterize cone responses in our analysis. Photoreceptor sensitivity functions, on the other hand, can be broadly categorized into ultraviolet, blue, green, and red according to the spectral range at which their response is maximum. Figure 1*b* shows the sensitivity functions of red, green, and blue zebrafish cones (Cornelius, 2021; Yoshimatsu et al., 2021b), which we use as a template for our main analysis. Nevertheless, we have also studied other sets of sensitivity curves, from different species, finding similar results (see, e.g., Fig. 4 in supplementary material). Zebrafish curves were obtained experimentally using LEDs ranging from 350 to 650 nm, with the same luminance (Yoshimatsu et al., 2021a). With these sensitivity functions and the Gaussian chromatic stimuli previously described, we use Equation 1 to model photoreceptor responses. As shown in Figure 1*d*, Gaussian chromatic stimuli with the same luminance intensity and far from the saturating regime yield responses similar to the sensitivity functions, but with a different overall intensity.

We propose a neuronal population model to characterize the average activity of cones and horizontal cells using the membrane potential, *h_i_*, of the embedded neurons. Currents induced by excitatory and inhibitory synaptic connections are proportional to the number of presynaptic neurons and their corresponding average membrane potential, such that the larger the number of synaptic connections, the stronger the induced internal current. In homogeneous populations, we can consider the magnitude of such internal currents to be a function of the average presynaptic membrane potential, *F*[*h_j_*], multiplied by a coupling constant, *w_ij_* (Gerstner et al., 2014). We therefore model the dynamics of these networks using the membrane potentials *h_i_*(*λ*) of neurons in the corresponding populations, that is,

(3) $$\tau_{i}\;\frac{\partial h_{i}}{\partial t}=-h_{i}+I_{i}+\sum_{j}w_{ij}F_{j}\left[h_{j}\right]\;i,j=\{R,G,B,H\},$$

with *τ_i_* the membrane potential time constant.

### Code accessibility

Codes, data, and supplementary material are available in the GitHub repository of the paper at the following link: https://github.com/luframirezoc/Chromatic_coding.

## Results

### Dichromatic system

In large neuronal systems, neurons with similar functional properties can be grouped into homogeneous populations described by their average behavior. Photoreceptors in dichromatic networks, for instance, can be grouped into two main populations characterized by their sensitivity function. Similarly, horizontal cells can be grouped into one or more populations that characterize photoreceptor responses. More specifically, we can think of the network shown in Figure 1*a* as a system composed of two populations, *R* and *G*, for red and green photoreceptors, and a single horizontal-cell population, *H*. Chemical synapses and gap junctions between neurons are lumped into effective interactions among populations that are either excitatory or inhibitory, depending on the presynaptic population.

As described in the previous section, we characterize the average activity of such dichromatic network with the membrane potentials, $h_{R}(\lambda ),\;h_{G}(\lambda )$ and $h_{H}(\lambda )$ of neurons in the corresponding populations, that is,

(4) $$\frac{\tau_{E}}{h_{0}}\;\frac{\partial h_{R}(\lambda )}{\partial t}=-\frac{h_{R}(\lambda )}{h_{0}}+I_{R}(\lambda )+w_{RG}\;F_{E}[h_{G}(\lambda )]+w_{RH}\;F_{I}[h_{H}(\lambda )]$$

(5) $$\frac{\tau_{E}}{h_{0}}\;\frac{\partial h_{G}(\lambda )}{\partial t}=-\frac{h_{G}(\lambda )}{h_{0}}+I_{G}(\lambda )+w_{GR}\;F_{E}[h_{R}(\lambda )]+w_{GH}\;F_{I}[h_{H}(\lambda )]$$

(6) $$\frac{\tau_{I}}{h_{0}}\;\frac{\partial h_{H}(\lambda )}{\partial t}=-\frac{h_{H}(\lambda )}{h_{0}}+w_{HR}\;F_{E}[h_{R}(\lambda )]+w_{HG}\;F_{E}[h_{G}(\lambda )],$$

with *h*_0_ the membrane potential when applying a unit of current (1 mA), that we set to unity without loss of generality(Gerstner et al., 2014). The last two terms on the right-hand sides of Equations 4 and 5 correspond to excitatory and inhibitory connections, respectively, with coupling parameters, *w_ij_*, which are positive for excitatory currents and negative otherwise; the first (second) subscript denotes the postsynaptic (presynaptic) population. The functions *F_I_*[⋅] and *F_E_*[⋅], characterize the population response to either inhibitory or excitatory currents, respectively. The second term, *I_i_*, corresponds to the magnitude of the independent photoreceptor response described by Equation 1. For simplicity, we refer to these terms as currents, but we remind the reader that they are dimensionless. Since horizontal cells do not receive direct external stimulation, the only source terms in Equation 6 correspond to the currents because of photoreceptor populations. The explicit dependence on the wavelength in Equations 4–6 characterizes chromatically diverse external stimuli; for simplicity, we omit this dependence in all subsequent equations.

The membrane time constants on the left-hand side of Equations 4–6, *τ_E_* for excitatory neurons and *τ_I_* for inhibitory neurons, introduce two time scales that are related to neuron responses and the latency of the feedback mechanism. Some experimental works (for review, see Chapot et al., 2017) show the existence of two fast feedback mechanisms from HCs; ephaptic and proton-mediated feedback, highlighting the suitability of HC for tasks involving fast adjustment of cone responses. We take it into consideration, assuming that the time membrane constant of inhibitory neurons is much faster than the time membrane constant of excitatory neurons, $\tau_{I}<<\tau_{E}$, which allows us to simplify Equations 4–6 as:

(7) $$\tau_{E}\;\frac{\partial h_{R}}{\partial t}=-h_{R}+I_{R}+w_{RH}F_{I}\left[\;\tanh\left(w_{HR}\;F_{E}[h_{R}]+w_{HG}\;F_{E}[h_{G}]\right)+1\;\right]+w_{RG}\;F_{E}[h_{G}]\tau_{E}\;\frac{\partial h_{G}}{\partial t}=-h_{G}+I_{G}+w_{HG}F_{I}\left[\;\tanh(w_{HR}\;F_{E}[h_{R}]+w_{HG}\;F_{E}[h_{G}])+1\;\right]+w_{GR}\;F_{E}[h_{R}]h_{H}=w_{HR}\;F_{E}[h_{R}]+w_{HG}\;F_{E}[h_{G}],$$

where we have set FI[h]=tanh hh + 1. This response function is always positive and saturates with strong stimulation, resembling typical cone activity (see supplementary material for further details on the response functions in the GitHub repository).

The experimental observations of zebrafish outer retinal layers lead us to ask whether inhibitory feedback mechanisms are advantageous for chromatic encoding. More generally, whether networks of Type I are more useful when compared with networks of Type II. We use the population model previously described to investigate this question first in dichromatic networks and subsequently in trichromatic ones. The set of Equation 7 provides a simplified framework to study the time evolution of population membrane potentials in dichromatic networks as a function of the synaptic strengths, allowing us to compare Type-I networks, with only inhibitory feedback, and Type-II networks, with strong intercone connections. Comparing the dynamics of the two types of networks should provide insights into encoding performance.

**Figure 2.**
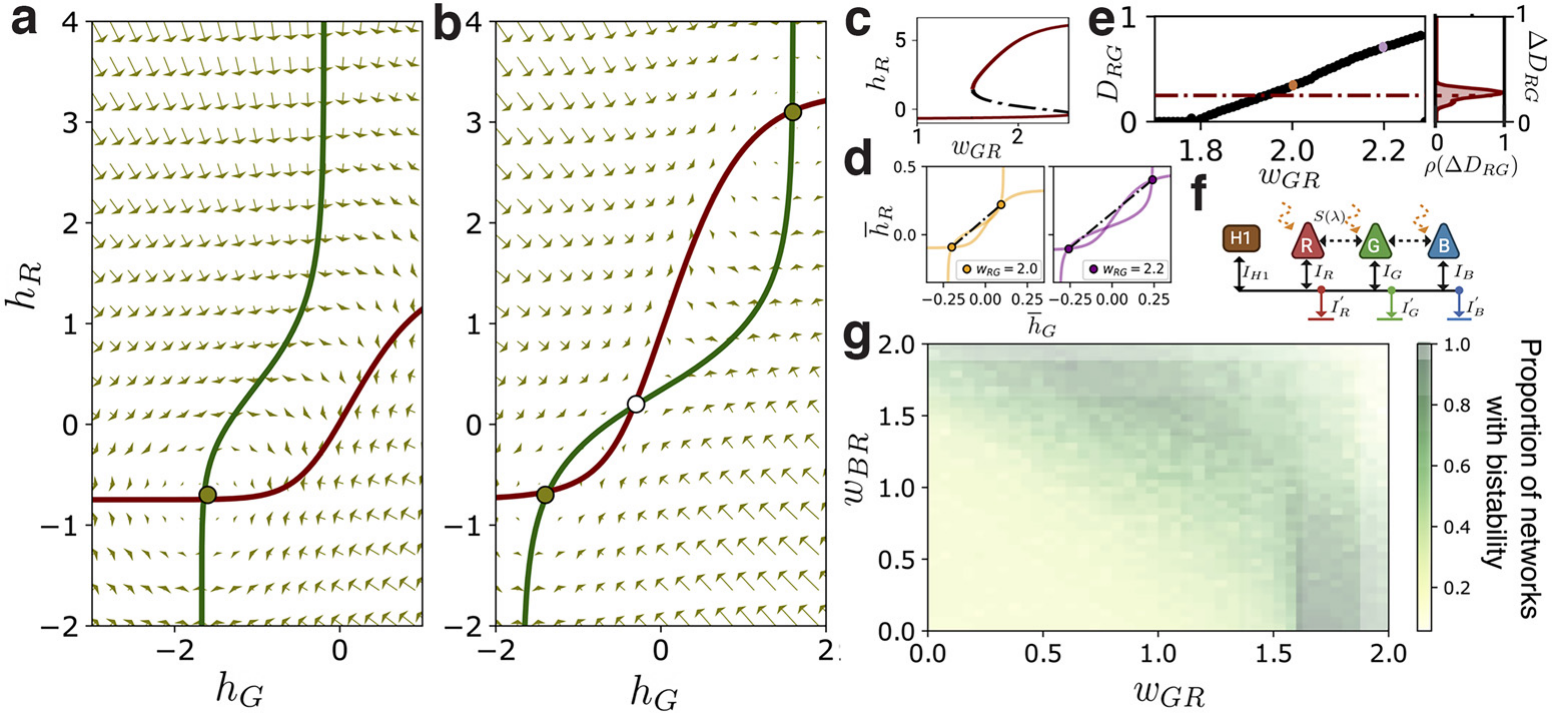
***a***, ***b***, Phase portraits of Equation 7 for a dichromatic network with red and green photoreceptors, and with the parameters $w_{HR}=1.5,w_{RH}=-1.7,\;w_{HG}=0.9,w_{GH}=-1.1,S(\lambda )=N(\lambda =380,1)$, and (***a***) $w_{GR}=w_{RG}=0$ corresponding to Type-I and (***b***) $w_{GR}=w_{RG}=1.8$, corresponding to a Type-II network. ***c***, Bifurcation diagram of *h_R_* for the intercone coupling parameters $w_{GR}=w_{RG}=[1,2.5]$. ***d***, Normalized phase portraits for red and green responses with two stable fixed points at a distance *D_RG_* (dashed line). The plot on the left (right) corresponds to the phase portrait of a Type-II network with the excitatory couplings $w_{RG}=w_{GR}=2.0$ ( $w_{RG}=w_{GR}=2.2$). ***e***, Distance *D_RG_* between stable fixed points in the phase plane as a function of the excitatory coupling; the parameters are the same as in ***c***. The distribution on the right corresponds the SD, $\Delta_{D_{RG}}$, obtained from zebrafish experimental data; the dashed red line is the mean error. ***f***, Trichromatic sketch of a fully connected network with an external stimulus, *S*(*λ*). R: red cones, G: green cones, B: blue cones, H: horizontal cells. Solid black arrows represent inhibitory synaptic connections on cones from horizontal cells. Dashed black arrows represent excitatory synaptic connections between cones. ***g***, Proportion of networks exhibiting multistability. Color intensity represents the normalized sum over the discrete interval *w_RG_* = [0.1, …, 2.0], for a fixed combination of the excitatory parameters, *w_GR_* and *w_BR_*. All points correspond to the same combination of inhibitory parameters used before.

A Type-I network is characterized by weak intercone connections, that is, $w_{RG}=w_{GR}\approx 0$ in Equation 7. This regime allows an analytic solution of the stationary state as a function of the four remaining coupling parameters, and all the chromatic stimuli illustrated in Figure 1*c*. Assuming a saturating excitatory response function, *F_E_*[*h*] = tanh (*h*)* *+* *1, We find that for any set of parameters, there is a unique stable fixed point in the two-dimensional domain $\left\{h_{r}\times h_{g},\;\in {\mathbb{R}}^{2}\right\}$. Figure 2*a* shows a typical phase portrait.

The existence of a single fixed point implies that the stationary response to a stimulus *S*(*λ*) is unique and independent of the initial state. Our results show that regardless of variations in the coupling strengths among neuronal populations, networks with only inhibitory feedback exhibit a unique response to a given chromatic stimulus, hence reliable encoding of chromatic stimuli. In the outermost retinal layers, this is desirable to avoid ambiguity in chromatic encoding and transmission.

**Figure 3.**
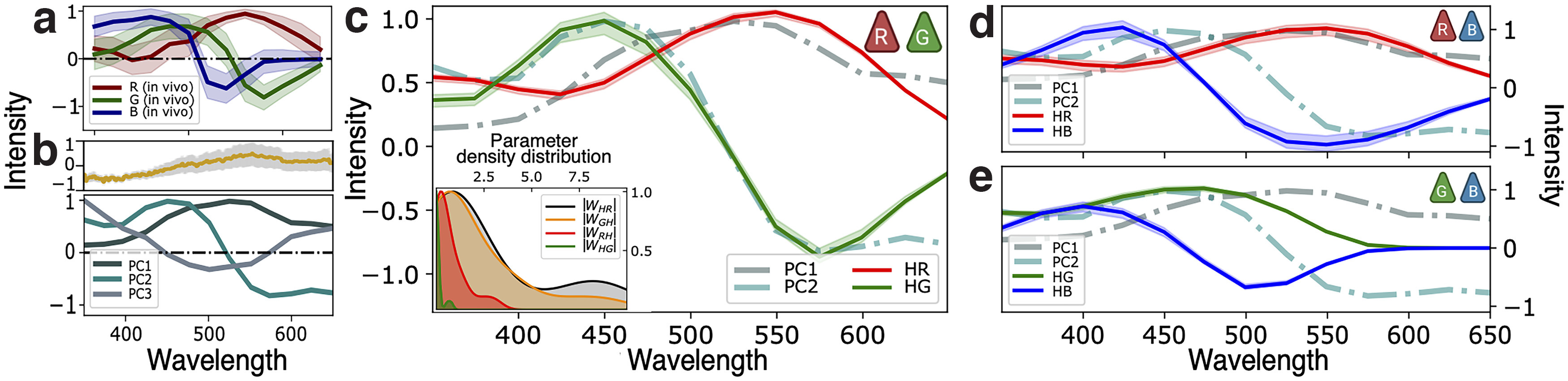
***a***, *In vivo* responses of red, green, and blue zebrafish opsins (Cornelius, 2021; Yoshimatsu et al., 2021b). ***b***, Upper panel, Hyperspectral data from 30 images of aquatic natural images typical of the zebrafish larvae environment (Cornelius, 2021; Yoshimatsu et al., 2021b). Lower panel, First three principal components obtained from the hyperspectral data. ***c–e***, Stationary solutions of Equation 7 for the optimal coupling parameters of a dichromatic network with (***a***) red and green, (***b***) red and blue, and (***c***) green and blue photoreceptors. Inset in ***a*** shows the distributions of the optimal parameters (absolute value, |*w_ij_*|) over 20 repetitions of the gradient descent algorithm starting from different initial values; inhibitory parameters *w_iH_* are negative by definition; we used a KDE method to infer the curves. Red, green, and blue curves correspond to the stationary solutions of the membrane potentials *h_r_*, *h_g_*, and *h_b_*, respectively. Dashed curves correspond to the principal component curves of ***b***.

We now ask whether such behavior persists in a Type-II network. We calculate the fixed points of Equation 7 by determining the intersections of the nullclines for all coupling parameters in the discrete space *w_ij_* ∈ [0.1, 0.2, …, 5.0) for excitatory and *w_ij_* ∈ (–5.0, …, –0.1, 0) for inhibitory parameters. As shown in Figures 2*b*,*c*, we find that, in contrast to Type-I networks, Type-II networks with strong intercone couplings (compared with HC-couplings), can exhibit three fixed points, two of them stable and one saddle node. This means that if the difference between stable network responses or fixed points is larger than the intrinsic network noise, the same chromatic stimulus leads to two different encodings, increasing the entropy. We characterize this difference via the distance, *D_ij_*, between normalized fixed points in the phase plane. More specifically, we normalize the network responses by the maximum fixed point value, ${\bar{h}}_{i}=h_{i}/\max\{h_{i}^{*1},h_{i}^{*2}\}$, and calculate the distance between normalized stable fixed points, making an average over different spectral stimuli in the visual range. Figure 2*d* shows the average distance (dashed lines) corresponding to the cases of $w_{RG}=w_{GR}=2.0$ (orange) and $w_{RG}=w_{GR}=2.0$ (purple). To estimate the typical network noise, we use the variance of *in vivo* responses of zebrafish cones (Yoshimatsu et al., 2021a; Fig. 3*a*). For instance, for the RG-dichromatic network, we can use the SD of red and green cone responses to estimate the uncertainty, $\Delta_{D_{RG}}$, over different chromatic stimuli. Figure 2*e* shows the distance *D_RG_* as a function of the excitatory parameter *w_RG_* in the RG-Type-II network; the red dashed line corresponds to the mean uncertainty over different stimuli and colored markers indicate the cases shown in Figure 2*c*. We see that the stronger the excitatory parameter, the larger the response difference compared with $\Delta_{D_{RG}}$, leading to an ambiguous chromatic signal. Networks with other photoreceptor combinations, such as red-blue and green-blue, were also studied. Such combinations lead to similar phase portraits and dynamical properties, reinforcing our general conclusion (see supplementary material in the GitHub repository).

Thus, we have shown that the responses of a two-photoreceptor system to a given chromatic stimulus are always independent of the initial state of the network if feedback is predominantly inhibitory, via horizontal cells. Conversely, including excitatory intercone interactions (e.g., via gap junctions) can lead to responses that depend on the initial state of the network. To investigate whether these results can be further generalized, next we investigate trichromatic networks, contrasting our findings with zebrafish experimental observations.

### Trichromatic system

The preceding stability results on networks with two populations of photoreceptors sensitive to different spectral ranges, allow extrapolation to other species with functionally similar dichromatic retinas. Generalizing these results to species with more photoreceptors, however, is not straightforward. We advance in this direction by investigating trichromatic systems, expanding considerably the diversity of color-vision systems. The trichromatic networks studied here include long-wavelength (*R*), middle-wavelength (*G*), and short-wavelength (*B*) photoreceptors, and a single horizontal cell population (H) that provides inhibitory feedback to cone populations (see Fig. 2*f*). Assuming that, as in the dichromatic network, $\tau_{I}<<\tau_{E}$, the equations of motion are as following:

(8) $$\tau_{E}\;\frac{\partial h_{i}}{\partial t}=-h_{i}+I_{i}+w_{iH}F_{I}\left[\sum_{j}w_{Hj}F_{E}[h_{j}]\right]+\sum_{j}w_{ij}F_{E}[h_{j}],i,j=\{R,G,B\},F_{E}[h]=F_{I}[h]\;=\;\tanh h+1.$$

We first investigate whether trichromatic networks with dominant inhibitory feedback (Type I) are also advantageous for chromatic encoding, as found in dichromatic networks. Similarly to the dichromatic case, the analytical solution of the system shows that Type-I networks exhibit a unique response to the same chromatic stimulus regardless of the coupling parameters strength. To investigate Type-II networks, we characterize the fixed points of Equation 8 for a discrete set of coupling combinations and for all the chromatic stimuli considered previously. In contrast to dichromatic networks, such fixed points, if any, are embedded in a three-dimensional phase portrait. To locate the fixed points, we minimize a cost function, L, that is zero if and only if ${\dot{h}}_{i}=0$ for all three photoreceptor populations in Equation 8, that is:

(9) $$L=\sum_{i}{\left(-h_{i}+I_{i}+w_{iH}F_{I}\left[\sum_{j}w_{Hj}F_{E}[h_{j}]\right]+\sum_{j}w_{ij}F_{E}[h_{j}]\right)}^{2}.$$

We use a gradient-descent algorithm to find the global minima of L for each parameter combination. It is easy to verify that, depending on the parameter combination, the network can exhibit one, two or three fixed points. Similarly to the dichromatic case, multiple fixed points are common in Type-II networks with strong intercone connections. To see this in detail, note that Equation 9 has six excitatory parameters corresponding to the couplings between red, green, and blue cone populations. Considering a symmetric interaction between populations, only three free parameters, *w_RG_* = *w_GR_*, *w_RB_* = *w_BR_*, and *w_GB_* = *w_BG_*, remain. For each combination of inhibitory couplings previously studied, we calculate the number of fixed points for all combinations of these three remaining excitatory couplings, finding that the stronger the excitatory parameters, the larger the likelihood of bistability. We summarize this result in the intensity plot of Figure 2*g*. For each fixed combination of the parameters *w_GB_* and *w_RB_*, we count the number of networks exhibiting multiple stable fixed points when varying the discrete values *w_RG_* = [0.1, 0.2 … 2]. We find that networks with at least one strong excitatory intercone coupling tend to exhibit multiple responses to the same chromatic stimulus. As in the dichromatic analysis, when the distance between fixed points in the RGB phase-space is greater than the mean response noise, the network exhibits ambiguous encoding of chromatic stimuli. This behavior is similar for all Gaussian stimuli studied.

We conclude that adding a third type of photoreceptor to the outermost retinal networks does not change our general conclusion regarding feedback mechanisms for chromatic encoding. In contrast, these results support the hypothesis that outer retinal networks with predominantly inhibitory feedback provide an advantage for reliable and unambiguous chromatic encoding, allowing a direct comparison with zebrafish experimental observations. As a complementary study, we investigated six other species with different sets of opsins (see supplementary material in the GitHub repository), finding similar dynamical properties. This suggests that our findings might be generalized to other species with similar outer retinal circuits.

### Efficient chromatic encoding

In the previous section, we focused on the synaptic strengths between populations that yield stable responses. In this section, we use both zebrafish *in vivo* photoreceptor activities (Yoshimatsu et al., 2021a) and hyperspectral data from zebrafish environments (Zimmermann et al., 2018) to investigate the architecture of outer retinal networks from the viewpoint of efficient encoding and transmission of chromatic information.

Figure 3*a* shows the *in vivo* spectral responses of zebrafish photoreceptors. Remarkably, interactions with horizontal cells cause green and blue neurons to exhibit opponent responses to chromatic stimuli, which, as discussed above, suggests early optimization of chromatic information encoding. Adopting this optimization hypothesis, we expect: (1) reduced redundancy of network responses in spectral space (Buchsbaum and Gottschalk, 1983); (2) efficient encoding of the environmentally available chromatic information. As a proxy of chromatic encoding, we use a principal component analysis of the hyperspectral data of aquatic naturalistic images typical of zebrafish environments studied previously (Zimmermann et al., 2018). In accord with analysis by Yoshimatsu et al. (2021a), we find that the first three principal components capture >97% of the chromatic data variance. Figure 3*b* shows these three principal components (PC1–PC3), the first without zero-crossings, the second with one zero-crossing, and the third with two. Comparing Figure 3*a*,*b*, we observe that the *in vivo* red and green cone responses match the first two principal components qualitatively, supporting the hypothesis of efficient encoding of chromatic stimuli in zebrafish outermost retinal layers.

We begin our analysis by studying the dichromatic network shown in Figure 1*a* and described by Equation 7. We adjust the coupling parameters, *w_ij_*, such that the spectral responses of the photoreceptor populations, *h_R_* and *h_G_*, match the first two principal components, which together explain >91% of the hyperspectral data variance. (We use the L-BFGS-B minimization algorithm to obtain the coupling parameters yielding the best match.) Figure 3*c* shows the solutions of Equation 7 for a network with only inhibitory feedback (in red and green), with both a no zero-crossing and a single zero-crossing curve, as expected for color-opponent signals (Hurvich and Jameson, 1955; Buchsbaum and Gottschalk, 1983). The inset shows the density of the coupling parameter absolute value $\left|w_{ij}\right|$ of Equation 7 over different basins of attraction. Similarly, we adjust the parameters to investigate a Type-II network with both inhibitory feedback and excitatory intercone connections; in all cases, optimization leads to weak or negligible excitatory couplings, producing results similar to those found in the inhibitory network. Type-II networks with imposed strong excitatory couplings might allow a fit of the first two principal components. Nevertheless, when compared with Type-I networks, they are disadvantageous since they are less reliable and increase metabolic cost unnecessarily.

The previous results show that optimized zebrafish-like dichromatic networks are Type-I since they do not require strong excitatory intercone couplings to match the two first environmental PCs. Together with the stability analysis, our findings suggest that zebrafish-like retinal networks provide unambiguous and efficient encoding of chromatic environmental information in the outer layer. This means that network responses are consistent (at least for $\tau_{I}<<\tau_{E}$) for any initial condition. We also studied other opsin combinations, leading to the results shown in Figures 3*d*,*e*. We observe that having only blue and red photoreceptors provides a qualitatively good fit to the first principal component, but not to the second. Moreover, dichromatic networks with only blue and green photoreceptors cannot fit either of the first two principal components. Contrasting the results for these two-photoreceptor combinations, we conclude that zebrafish-like dichromatic networks with long -and middle-range photoreceptors have the best performance when encoding chromatic information typical of the zebrafish environment.

**Figure 4.**
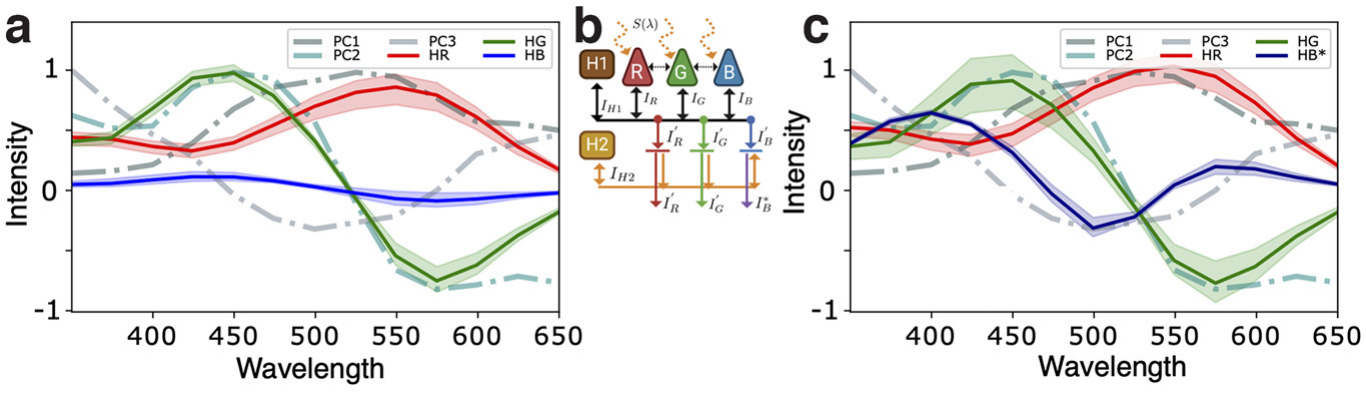
***a***, Stationary solutions of Equation 8 for the optimal coupling parameters of the trichromatic network sketched in the inset. ***b***, Sketch of a fully connected trichromatic network with two types of horizontal cells providing two successive inhibitory feedback mechanisms. ***c***, Stationary solutions of Equations 8 and 10 for the optimal coupling parameters of the trichromatic network sketched in the inset. Red, green, and blue curves correspond to the stationary solutions of the membrane potentials *h_r_*, *h_g_*, and *h_b_*, respectively. Dashed curves correspond to the principal component curves of Figure 3*b*.

Next, we analyze trichromatic networks, restricting the parameter space to reproduce the expected opponent responses, as we have done for dichromatic networks. Following the same procedure as before, we fit the membrane potential response of each photoreceptor population, *h_R_*, *h_G_*, and *h_B_*, to the first three principal components of the naturalistic images. As shown in Figure 4*a*, the optimized network yields a poor fit to the third principal component, and the network responses do not match qualitatively the expected responses. Instead, we find a single zero-crossing in the blue photoreceptor response curve. We note, however, that *in vivo* recordings of photoreceptor responses do not match this third principal component either, as shown in Figure 3*a*.

For trichromatic systems, Type-I and Type-II networks are unable to reproduce all three principal components, leading us to ask whether an expanded network, e.g., with a second parallel inhibitory feedback or with a fourth type of photoreceptor is capable of realizing this task. We begin by including a second type of horizontal cell, H2, providing feedback to only two of the three cone populations. Repeating the previous analyses, we find that networks with two parallel inhibitory feedback mechanisms do not eliminate the errors in fitting of the third principal component. Similarly, we investigated whether a fourth type of photoreceptor, with a maximum response in the UV range, might allow the other three photoreceptor responses to match all three principal components simultaneously. We found that adding such a UV cone population has little effect on the functional responses of the other cones, in agreement with the experimental analysis of zebrafish (Yoshimatsu et al., 2021a; see supplementary material in the GitHub repository).

From these results we conclude that in zebrafish retinas, trichromatic networks are incompatible with obtaining the first three principal components of environmental chromatic data at the first synaptic connection. This motivates us to add a second synaptic connection, or network layer, to verify whether such a network can be optimized to reproduce the expected results. Figure 4*b* shows a sketch of such a network, with a second type of horizontal cell, H2^*^, integrating the first layer responses, and providing feedback only to population B, which is the one unable to fit the third principal component. The blue cone population response, $h_{B}^{*}$, at the second layer is described by the following equation:

(10) $$\frac{\partial h_{B}^{*}}{\partial t}=-h_{B}^{*}+h_{B}+w_{H_{2}^{*}R}\;h_{R}+w_{H_{2}^{*}G}\;h_{G}.$$

with $w_{H_{2}^{*}R},w_{H_{2}^{*}G}<0$. Figure 4*c* shows the responses of the more efficient network after learning the parameters of Equations 8 and 10. The improved agreement between response curves and PCs suggests that obtaining the three efficient chromatic channels requires a second synaptic contact (or network layer), including a second type of inhibitory neurons. Although we modeled this population as a second type of horizontal neuron, other neuronal populations in inner retinal layers, such as bipolar cells, might also realize this task. We also stress that the stability analysis does not change since the excitatory couplings remain weak, which leads to a reliable and efficient network to encode chromatic information from zebrafish environment.

In summary, adopting ideas from information theory, chromatic opponency and network stability, we have shown that after the first synaptic connection, dichromatic networks can yield efficient responses when red cones are available. Otherwise, responses of photoreceptor populations are suboptimal. Generalizing to trichromatic networks, we find that, after the first synaptic connection, photoreceptors are unable to exhibit all three opponent responses. Only by including a second synaptic connection, or network layer, with a fourth neuronal population, can photoreceptor responses match the PCs.

#### Optimal sensitivity peaks

Our study identified networks that optimize the encoding of chromatic information available in the zebrafish environment, using the set of zebrafish sensitivity curves. Now we ask whether such curves are optimal for the network to fit the given chromatic information, or if instead, there are other opsin combinations leading to more precise chromatic encoding. Although optimization is not the only feature that determines visual system properties, it has been shown that some species adapt aspects, such as the sensitivity functions, to fit the environmental conditions (Chittka and Menzel, 1992; Fasick and Robinson, 2000).

**Figure 5.**
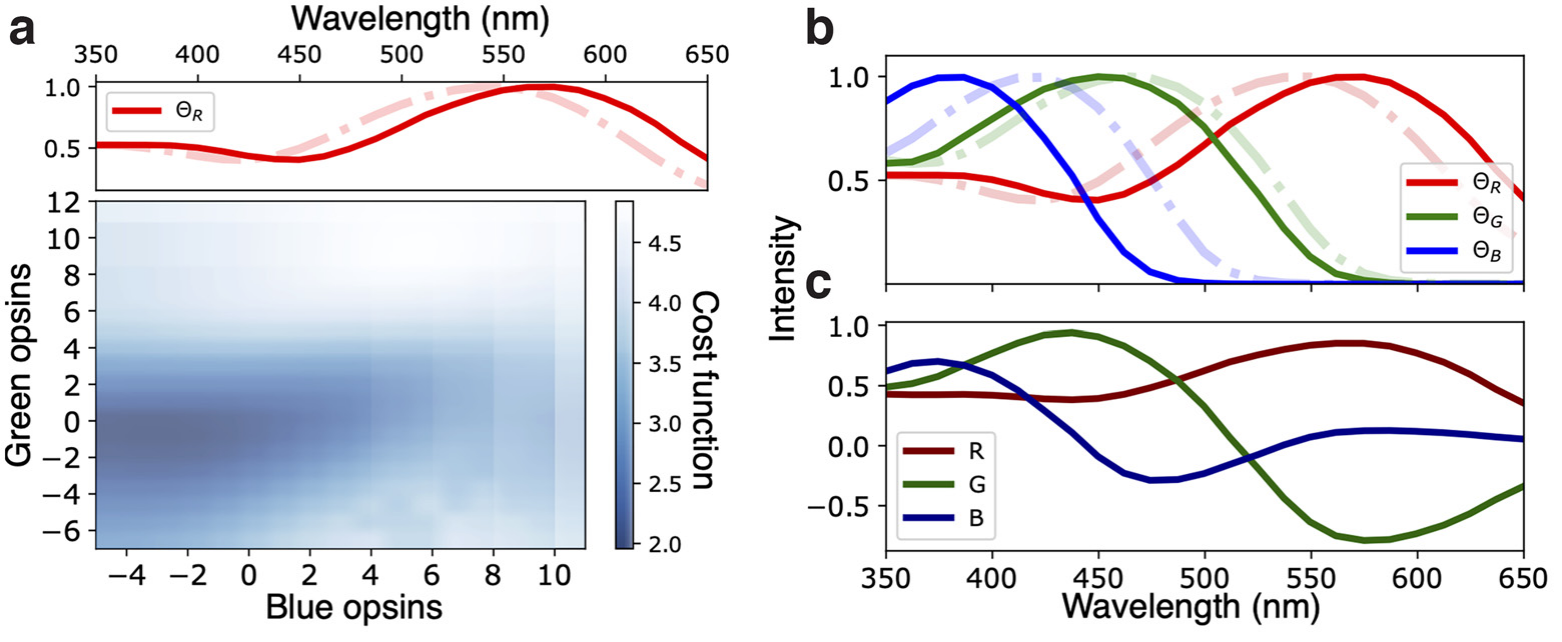
***a***, Intensity plot of the cost function (Eq. 11) for different opsin curves combinations, darker colors represent smaller values. Red opsin was first fixed to optimize all possible combinations; the optimal curve is shown in the upper plot; x and y labels in the intensity plot represent the number of shifts (in steps of 5 nm) of blue and green curves respectively, with the sign indicating the shift direction. ***b***, Comparison between experimentally observed sensitivity functions (dashed lines) and the optimal fitting curves (solid lines). Arrows indicate the optimal shift direction. ***c***, Network responses for this optimal set of opins.

To answer this question, we contrast the performance of networks composed of different opsin combinations obtained by varying the wavelength of maximum sensitivity of zebrafish cones, while maintaining the shape of the distribution fixed (we use a discrete step interval of Δ*_λ_* ≈ 12 nm). Variations of response-curve shapes are being investigated in another work contrasting oil-droplet effects in color encoding. For all opsin combinations, we calculate the optimal two-layer network, described by Equations 8 and 10, fitting the first three principal components of the zebrafish environment, as in the previous sections. To quantify the network encoding performance, we define the cost function as follows:

(11) $$C_{o}^{2}={\left(h_{R}-PC_{1}\right)}^{2}+{\left(h_{G}-PC_{2}\right)}^{2}+{\left(h_{B}^{*}-PC_{3}\right)}^{2}.$$

such that near-zero values correspond to optimal performance. Using the same interval Δ*_λ_* for blue and green opsins, we calculate the cost function for all possible combinations within the interval (350, 650) nm, and search for an optimal set of opsins. Figure 5*a* shows an example of the intensity plot of the cost function for all green and blue opsin combinations given a fixed red sensitivity curve. We find that the global minimum, over all combinations of the three curves, is reached at $\Theta_{R}^{*}(\lambda )=\Theta_{R}(\lambda +2\Delta_{\lambda }),\;\Theta_{G}^{*}(\lambda )=\Theta_{G}(\lambda -\Delta_{\lambda })$ and $\Theta_{B}^{*}(\lambda )=\Theta_{B}(\lambda -3\Delta_{\lambda })$, as illustrated in Figure 5*b*,*c*.

From this optimization analysis, we conclude that compared with zebrafish, the optimal set of cones improves performance by ∼13%, suggesting that retinal networks with zebrafish cones are quite efficient to encode chromatic information. Other strategies, such as shrinking of the sensitivity curves, might provide further optimization, but as previously mentioned, we lead that discussion to another work. Extrapolating these results to other species with similar environmental chromatic conditions and retinal circuitry requires knowledge of the independent cone responses, or sensitivity curves to properly compare the networks. Studies involving species with different environmental conditions would naturally require analysis of the corresponding environmental hyperspectral data.

## Discussion

Identifying the retinal circuits specialized in chromatic discrimination leads to an understanding of how organisms extract spectral information from their environment (Huang and Rao, 2011; Baden and Osorio, 2019). *In vivo* experiments on outer retinal circuits, however, are only feasible in a restricted number of species, limiting a broader study. In this situation, theoretical models are useful to predict general features that can be tested in such model organisms and extrapolated to others beyond experimental access. Our study attempts to identify features of zebrafish-inspired outer retinal networks to understand more broadly the biological fundamentals of chromatic encoding and transmission in visual systems. As mentioned earlier, we focused our work on networks that mediate chromatic information via HCs. Other species, such as butterflies (Belušič et al., 2021), with different network architectures will be studied in future works.

In the first part of our study, we found that in outer retinal networks with fast inhibitory feedback, intercone excitatory connections can lead to bistability, so that network responses to a given chromatic stimulus could be ambiguous. Ideally, one expects photoreceptors to encode as much relevant visual information as possible from the environment and transmit it reliably to downstream circuits. More complex tasks, such as edge or movement detection, for which such bistability might be desirable, are expected to take place later in the visual system. Our results suggest that, in retinas with circuitry similar to that of zebrafish, intercone excitatory couplings, if present, are likely not involved in chromatic encoding and/or are much weaker than inhibitory feedback. Some experimental studies on macaques and other vertebrate species (Raviola and Gilula, 1973; DeVries et al., 2002; Laughlin, 2002) have shown evidence of both cone-cone and cone-rod gap junctions in the foveal region. The role of such connections, however, is not fully understood. In ground squirrel retinas, for instance, such connections seem to play an important role in increasing the signal-to-noise ratio (DeVries et al., 2002); similar behavior has been found in macaque retinas (Tsukamoto et al., 1992). Noise reduction is highly relevant in low-luminance environments. At high luminance, where color discrimination is possible, the effects of gap junctions seem to be negligible given their weak extent, supporting the hypothesis that gap junctions are involved in achromatic tasks. Some other works (Jackman et al., 2011) have shown evidence of excitatory feedback in individual synapses between HCs and cones, which might lead to other effective connections, not considered in our model.

In addition to studying different types of feedback, we investigated network architectures leading to efficient encoding of the available chromatic information. Determining whether outer retinal circuits are optimized to encode available chromatic information allows one to gauge the relevance of color discrimination in a given species. Such findings might afford insights into other areas, such as ecology and animal behavior. As suggested previously (Yoshimatsu et al., 2021a), zebrafish seem to encode of the chromatic information efficiently, qualitatively matching the first two PCs of the hyperspectral data. We formalize these ideas in a neuronal population model. Specifically, we find that a network with two interneuron layers is necessary to fit reliably the three first PCs. We would expect retinas to optimize the trade-off between information transmission and metabolic cost, such that information transmission is guaranteed at the lowest energy expenditure. Nevertheless, even with an optimal architecture, more complex networks are necessary to transmit specific information, as suggested by our results. Whether it is advantageous for an organism to invest energy in an additional retinal layer to improve chromatic discrimination depends on species-specific interactions with its habitat. These results are general for zebrafish-like networks with inhibitory interneuronal layers and similar environmental conditions.

Our study of network architecture was performed using zebrafish opsin curves as a reference. Subsequently, we investigated whether other opsin combinations might lead to further optimization of the network, that is, whether it would be possible to improve zebrafish performance at encoding environmental chromatic information. We find that optimizing opsin combinations, by varying their wavelength peak, leads to an improvement of ∼13% in zebrafish performance. As mentioned earlier, in some species, such as bees, photoreceptor sensitivity functions are highly tuned to the environmental chromatic spectrum, leading to an efficient encoding of color information (Chittka and Menzel, 1992; Fasick and Robinson, 2000). Our results on zebrafish show that they have an efficient chromatic encoding, but still there are other networks yielding modest improvements. Other relevant features, such as movement or edge detection, might favor retinal circuits that optimize other general aspects of visual stimuli, by penalizing slightly chromatic information. As before, these results hold for zebrafish-inspired retinal circuits under similar environmental conditions.

## Synthesis

Reviewing Editor: Nicholas J. Priebe, University of Texas at Austin Decisions are customarily a result of the Reviewing Editor and the peer reviewers coming together and discussing their recommendations until a consensus is reached. When revisions are invited, a fact-based synthesis statement explaining their decision and outlining what is needed to prepare a revision will be listed below. The following reviewer(s) agreed to reveal their identity: NONE.

SYNTHESIS

Two reviewers evaluated the manuscript “Data-driven models of efficient chromatic coding in the outer retina” and both reviewers agree that the authors provide compelling evidence that the synaptic interactions and coding of the zebrafish retina is near optimal for the environment in which zebrafish live. The authors explore how inhibitory interactions between cones (via horizontal cells) produces responses characterized by a single fixed point, and thus generating responses in which a specific chromatic signal will generate a reliable and fixed response independent of the initial condition. In addition, the authors note that networks composed of excitatory interactions between cones can yield networks solutions in which there is more than one stable solution, and therefore that the initial condition could influence the activation pattern to a given spectral stimulus. The authors then explore how efficient the cone sensitivities of zebrafish are at encoding their environment. Overall, the manuscript provides an intriguing analysis of cone responses and represents an important step forward. There was a consensus among the reviewers that a few issues should be addressed.

1) Clarity. There are two points where the message that the authors are trying to convey is obscure. First, the discussion of the distance error (D_RG) on page 6 could be more clearly described. It might be helpful to show 2 different architectures with different degrees of excitatory interactions, or to indicate precisely in a figure the definition of the distance measurement. Second, in the section discussing the efficiency of the chromatic encoding, the authors conclude on page 9 that the chromatic networks are type I because they do not require strong excitatory inter-cone couplings to match the first two PCs. But this logic seems opaque to me since the authors do not show the type II networks could not also match the first 2 PCs as well.

2) What happens when there are both inhibitory interactions via the horizontal cells and excitatory coupling?

3) Minor Issues

a) Page 3 “to characterize cone responses in our analysis” should end with a period

b) Figure 1a, the dashed lines are not visible.

## Author Response

Major comments

The discussion of the distance error (DRG) on page 6 could be more clearly described. It might be helpful to show 2 different architectures with different degrees of excitatory interactions, or to indicate precisely in a figure the definition of the distance measurement.

We apologize for the lack of clarity about this point. The distance DRG shown in Fig. 2e (in the reviewed manuscript) corresponds to the same type of network architecture and different excitatory couplings. We adapted suggestion 2 by including a new subfigure (2d) illustrating the definition of DRG. Figure 2d shows the phase portrait of two type-II networks with different excitatory couplings, indicating the distance between fixed points. In addition, we have added some text (bold text in the paragraph of line 209) regarding the definition of the distance.

1. In the section discussing the efficiency of the chromatic encoding, the authors conclude on page 9 that the chromatic networks are type I because they do not require strong excitatory inter-cone couplings to match the first two pc. But, this logic seems opaque to me since the authors fo not show the type II networks could not also match the two pc as well.

2. What happens when there are both inhibitory interactions via horizontal cells and excitatory couplings?

We apologize for the lack of clarity about this point. In Sections 5.1 and 5.2, we show that Type-I networks are more advantageous than Type-II networks since they allow an unambiguous encoding of color information. In section 5.3, we begin by investigated

Type-I networks, showing that they can fit the first two principal components. investigating Type-II networks, we find that the optimization process leads to networks with weak or negligible excitatory couplings, similar to Type I networks. This analysis leads us to conclude that optimized zebrafish-like networks are Type-I, in accordance with the experimental observations. In addition, the additional inter-cone connections might increase unnecessarily the metabolic cost compared to type-I networks.

In the paragraphs of lines 307 and 323, we have added some text further explaining these aspects: (...) Similarly, we adjust the parameters to investigate a Type-II network with both inhibitory feedback and excitatory inter-cone connections; in all cases, optimization leads to weak or negligible excitatory couplings, producing results similar to those found in the inhibitory network. Type-II networks with imposed strong excitatory couplings might allow a fit of the first two principal components. Nevertheless, when compared to Type-I networks, they are disadvantageous since they are less reliable and increase metabolic cost unnecessarily.

The previous results show that optimized zebrafish-like dichromatic networks are Type-I since they do not require strong excitatory inter-cone couplings to match the two first environmental PCs. Together with the stability analysis, our findings suggest that zebrafish-like retinal networks provide unambiguous and efficient encoding of chromatic environmental information in the outer layer (...)

Minor comments

1. Page 3 to characterize cone responses in our analysis should end with a period.

2. Fig. 1a, the dashed lines are not visible.

We apologize for having overlooked these points.

We have implemented the corrections and modifications suggested.
